# Association Between *JAK2* V617F Somatic Mutation and Thoracic Aortic Aneurysms

**DOI:** 10.3390/genes17040364

**Published:** 2026-03-24

**Authors:** Simon Collins, Mohammad A. Zafar, John A. Elefteriades

**Affiliations:** Aortic Institute at Yale New Haven Hospital, Yale School of Medicine, New Haven, CT 06511, USA; simon.collins@yale.edu (S.C.); mohammad.zafar@yale.edu (M.A.Z.)

**Keywords:** thoracic aortic aneurysm (TAA), *JAK2* V617F, variant allele frequency (VAF), abdominal aortic aneurysm (AAA), myeloproliferative neoplasms (MPNs), clonal hematopoiesis of intermediate potential (CHIP)

## Abstract

**Background/Objectives:** Thoracic aortic aneurysms have long been associated with germline mutations such as *FBN1*, *TGFBR2*, and *COL3A1*, which predispose to Marfan, Loeys–Dietz, and Ehlers–Danlos syndromes, respectively. However, recent research has identified a correlation between the *JAK2* V617F somatic mutation and thoracic aortic aneurysm formation. This review aims to synthesize the current evidence on the relationship between *JAK2* V617F and TAA development. **Methods:** A literature review was conducted using PubMed reviewed articles up to June 2025. Search terms included “thoracic aortic aneurysm”, “somatic mutations” and “*JAK2* V617F”. Relevant clinical datasets and population-based cohort studies were identified and evaluated. **Results:** The available studies demonstrated a consistent association between *JAK2* V617F and thoracic aortic aneurysm formation, with *JAK2* V617F variant allele frequency (VAF) being a valuable biomarker of aneurysm risk. The mutation is accompanied by the onset of increased cytokine production, pro-inflammatory leukocytes, and elevated expression levels of MMPs—all of which drive elastin degradation and are classically associated with thoracic aortic aneurysm development. **Conclusions:** Compelling emerging evidence supports an association between the *JAK2* V617F somatic mutation and the formation of thoracic aortic aneurysms, with VAF acting as a valuable biomarker for aneurysm risk. However, no studies have evaluated whether increasing VAF influences aneurysm growth rate, highlighting the need for future clinical research.

## 1. Introduction

Thoracic aortic aneurysm (TAA) is a life-threatening condition characterized by progressive dilation of the thoracic aorta, often asymptomatic until occurrence of catastrophic dissection or rupture [1]. TAA is characterized by the degenerative remodeling of the aortic medial extracellular matrix, often caused by a genetic predisposition resulting in increased activity of matrix metalloproteinases (MMPs), which weakens the aortic wall and predisposes the individual to progressive dilation [2].

Myeloproliferative neoplasms (MPNs) are a group of blood cancers characterized by overproduction of mature red blood cells, white blood cells, or platelets in the bone marrow [3]. The most common forms of MPNs are polycythemia vera (PV), essential thrombocythemia (ET), and primary myelofibrosis (PMF) [3]. These clonal hematologic malignancies are driven by the presence of an acquired somatic mutation [4]. A somatic mutation is a genetic alteration that occurs in non-germline (somatic) cells after conception and is therefore acquired over a person’s lifetime [5]. The most common driver mutation of MPNs occurs on the *Janus Kinase* (*JAK*) *2* gene. *JAK2* encodes a protein kinase that is a part of the JAK-STAT pathway, which regulates growth and development of blood cells. The specific mutation in the *JAK2* gene in TAA occurs at codon 617 and involves the substitution of a valine for phenylalanine, and it is called *JAK2* V617F [4,6]. The quantifiable abundance of *JAK2* V617F within an individual is given by a metric called variant allele frequency (VAF). VAF represents the percentage of variant sequencing reads within a genetic locus [7]. The VAF of *JAK2* V617F varies by MPN subtype, as shown in Table 1 [8].

Clonal hematopoiesis of intermediate potential (CHIP) refers to a somatic mutation that causes the proliferation of pro-inflammatory immune cells within an individual’s bone marrow [9]. Studies have linked CHIP as a precursor to both hematologic malignancies and different cardiovascular diseases, such as atherosclerosis, heart failure, and aortic aneurysms. Commonly observed mutations include *DNMT3A*, *TET2*, *ASXL1*, and *JAK2* V617F, which alter hematopoietic stem cells and promote inflammation. The prevalence of CHIP increases as a person ages, with 10–20% of individuals older than 70 carrying such mutations [10]. Although CHIP is primarily considered an age-related phenomenon, additional environmental exposure may influence the expansion and persistence of specific mutant clones. Cigarette smoke exposure has been shown to promote the clonal expansion of hematopoietic cells harboring mutations such as *JAK2* V617F and *TET2*. A murine model demonstrated that cigarette smoke exposure accelerated the expansion of *JAK2* V617F and *Tet2*–/– clones, suggesting that environmental stressors may contribute to clonal expansion [11]. CHIP is often characterized by a mutant VAF of ≥2% [10]. To detect CHIP, blood tests and next-generation sequencing (NGS) are utilized and serve as valuable biomarkers allowing for early detection for at-risk individuals [12].

Germline mutations are a well-established and extensively studied cause of aortic aneurysms (for example, Marfan Syndrome, Loeys–Dietz Syndrome, and Ehlers–Danlos syndrome, as well as many non-syndromic mutations that affect only or predominantly the thoracic aorta, without systemic manifestations) [13]. Conversely, the role of somatic mutations in aortic aneurysm development remains far less explored. Over recent years, studies have detected an association between *JAK2* V617F and the development of aortic aneurysms [14,15,16,17,18]. Although this review focuses on the *JAK2* V617F mutation, it is possible that additional somatic mosaic variants may also contribute to TAA susceptibility. However, to our knowledge, there is currently no evidence supporting a significant role of such variants in TAA pathogenesis. This review examines these studies in a chronological order, so as to demonstrate the progress that has been accomplished in the understanding of this mutant gene and its role in aortic aneurysm formation.

The first two studies employed a translational design, combining germline mouse models that express *JAK2* V617F with clinical data from human patients to investigate the mutation’s role in the development of aortic aneurysms. Mouse models likely overestimate the impact of the *JAK2* V617F mutation compared with its effects in humans, due to direct iatrogenic germline engineering. However, the mechanisms of causation of aortic aneurysms via *JAK2* V617F mutations in mice are directly relevant to human aortic aneurysms and will be examined in the latter half of this communication.

## 2. Clinical Studies

The first study, entitled Crucial role of hematopoietic *JAK2* V617F in the development of aortic aneurysms, was published by a Japanese research group in 2021. The investigators examined 39 patients (age: 68 ± 12; 46% male) with MPNs harboring the *JAK2* V617F mutation, finding that 9 individuals (23%) had aortic aneurysms. Among these, seven had TAA and two had abdominal aortic aneurysm (AAA). Additionally, the VAF was reported as 51 ± 30%. However, VAF was not stratified in the analysis, so the potential effect of different allele burdens on clinical outcomes could not be assessed. This study also revealed increased expression levels of MMPs, cytokines, and circulating leukocytes, all of which are associated with aortic aneurysms [14].

The second study, entitled *JAK2* V617F mutation drives vascular resident macrophages toward a pathogenic phenotype and promotes dissecting aortic aneurysm, was published in 2022 by a French research group. The study recruited 382 *JAK2* V617F-positive patients and identified 157 (age: 65 ± 14; 61% male) who had had a recent body CT scan. While the exact number of individuals with aortic aneurysms was not specified, the investigators reported significant dilation of both the ascending and descending thoracic aorta, but not the abdominal aorta. Of note, all 157 imaged patients had a clinical diagnosis of an MPN. VAF and other clinical risk factors associated with an increased likelihood of aortic aneurysms were not reported [15].

The third study, entitled Increased Risk of Thoracic Aortic Aneurysms with *JAK2* V617F, was published in 2024. Using a UK Biobank blood-based whole-exome sequencing dataset of 417,759 participants, the investigators identified 118 individuals (age: 60.6 ± 6.5; 56.8% male) carrying the *JAK2* V617F mutation. Individuals with known hematologic malignancies or MPNs were excluded, so as to focus specifically on the contribution of CHIP to thoracic aortopathy. The VAF was reported as 33 ± 15%. The exact number of patients with aortic aneurysms was not reported. Instead, the association between *JAK2* V617F and TAA was quantified with a hazard ratio (HR) of 12.8 and *p* = 3.6 × 10^−7^. By contrast, the relationship between *JAK2* V617F and AAA was not significant, with a hazard ratio of only 1.4 and *p* = 0.76. To further confirm the robustness of the relationship between *JAK2* V617F CHIP and aortic aneurysm formation, the investigators excluded individuals with at least one abnormal blood cell count and repeated the statistical analysis. The association between *JAK2* V617F and TAA remained significant (HR 16.3, *p* = 8.1 × 10^−5^), while AAA formation remained insignificant [16].

The fourth study, titled Somatic Variants Acquired Later in Life Associated with Thoracic Aortic Aneurysms: *JAK2* V617F, by our group, was also published in 2024. We analyzed 12,349 clinical-grade exomes from the internal database of exome sequencing in the Yale DNA Diagnostics Lab. Among these, 12 individuals were positive for the *JAK2* V617F mutation. In this cohort, five patients (mean age: 70; 40% male) presented with TAA, with four being localized to the ascending aorta and one to the descending aorta. The VAF of these patients ranged from 11.2 to 20%. Due to the small sample size, no statistically significant association could be established between *JAK2* V617F and TAA [17].

The fifth study, titled Aortic Aneurysm Risk and Somatic *JAK2* V617F Variation: Insights From a Multicenter, Population-Based Cardiovascular Screening Study, was published by a research team from Denmark in 2025. The study utilized data from the DANCAVAS I and II trials, which included 8056 individuals screened for *JAK2* V617F. Of the 8056 individuals, 570 (7.1%) were positive for the mutation. Within this *JAK2* V617F-positive subgroup, the number of individuals presenting with TAA or AAA was 83 (14.6%) and 39 (6.8%) respectively. The researchers further stratified TAA into ascending and descending segments of the aorta, identifying 57 ascending and 26 descending aneurysms. In contradistinction to the previously examined studies, these researchers focused on the role that *JAK2* V617F VAF exerted on aneurysm formation. VAF was categorized into three groups: <1%, ≥1%, and ≥2%. Among the *JAK2* V617F-positive participants with VAF <1%, 44 out of 491 (9%) had ascending aortic aneurysms. Among those individuals with VAF ≥1%, 13 out of 79 (16.5%) had ascending aortic aneurysms. Finally, among those individuals who had a VAF ≥2%, 9 out of 56 (16.1%) had ascending aortic aneurysms. A positive association between higher VAF and ascending aortic aneurysm prevalence was observed, with an 11% increase in risk for each doubling of VAF. However, this relationship did not hold true with descending and abdominal aortic aneurysms. To test the relationship between *JAK2* V617F VAF and aortic aneurysms, the researchers applied both a univariate logistic regression model and a multivariate logistic regression model to each of the three sections of the aorta. For the ascending aorta both models showed significance with adjusted odds ratios (aORs) of 1.4 for VAF <1%, 2.7 for VAF ≥1%, and 2.5 for VAF ≥2%. However, when the multivariate logistic regression model was applied to the relationship between *JAK2* V617F VAF and descending/abdominal aortic aneurysms no significance was found [18].

When comparing these studies (see Table 2), several consistent trends emerge despite differences in study design and population. One of the most prominent findings is that the *JAK2* V617F mutation is consistently associated with aneurysm formation localized to the thoracic aorta, specifically the ascending aorta, with no definitive link observed in AAA. Although each study reports this relationship differently (from hazard ratio to actual case counts), they all converge to the same conclusion. Although the ascending and descending aortas are known to be very different organs, the regional specificity of *JAK2* V617F-driven vascular disease remains an understudied area, and the underlying mechanisms responsible for its ascending thoracic predominance are yet to be determined [19].

Another notable similarity across studies is the frequent presence of a diagnosed MPN in individuals who had the *JAK2* V617F mutation and TAA. While this association may not be unexpected, given that the *JAK2* V617F mutation is the most common driver mutation in individuals with MPNs, it remains a clinically significant and consistently reported finding. This recurring pattern raises an important question: Should patients with a diagnosed MPN be routinely screened for TAA?

Age also emerges as a shared characteristic, with most individuals carrying the *JAK2* V617F mutation and presenting with TAA being older adults [14,15,16,17,18]. Across the studies, the average age of these individuals was approximately 65 [14,15,16,17,18]. Given the somatic nature of the mutation and its accumulation with age, this finding is expected. It also aligns with what is known about CHIP, which becomes increasingly common in aging populations and can precede hematologic malignancy [20]. From a clinical perspective, this age group may already be undergoing CT, ECHO, or MRI scans for other comorbidities, creating a valuable window for TAA identification. Given the often-silent progression of TAA, particularly in older adults, routine imaging may help identify aneurysms that would otherwise go undetected.

Despite several shared characteristics, the studies also differ in key aspects that may influence their conclusions. One area where the studies diverged was in the reported VAF of the individuals with *JAK2* V617F and TAA. Taken together, the studies presented a wide range of *JAK2* V617F VAF, from a median of 4.6% in the Danish study to a mean of 16.5% in the Yale study to 51% from the Japanese investigation [14,17,18]. The observed difference in VAF is likely attributable to the populations that were studied. Certain studies focused on patients who had MPNs, while others excluded those individuals and prioritized the relationship of CHIP with aortopathy. The distinction between CHIP and MPN populations is important, as individuals with *JAK2* V617F CHIP typically have lower VAFs than those with diagnosed MPN [21]. Although individuals with *JAK2* V617F clonal hematopoiesis are at higher risk of developing hematologic malignancies, this progression does not always occur [22,23]. Hence, it is valuable that studies examine both cohorts: patients with *JAK2* V617F CHIP might account for unexplained cases of TAA, while patients with known MPNs might be at increased risk of TAA.

Additionally, the differences in population demographics is important. The studies that were reviewed come from around the world, with subjects of different races and ethnicities, which potentially contributed to the variability in the findings. Although the mean age difference among cohorts was modest, age may still contribute to variability in findings, given the age-dependent nature of both *JAK2* V617F clonal expansion and TAA (more frequent in older adults) [20]. The gender composition varied notably across the studies, with the proportion of male participants ranging from 40% to 90% [14,15,16,17,18]. While the male sex is a known risk factor for TAA, the specific role of sex in the vascular effects of *JAK2* V617F is unclear [24].

## 3. Animal Studies

The present section focuses on the murine models used in the two translational studies (Yokokawa and Al-Rifai).

**Figure 1:**
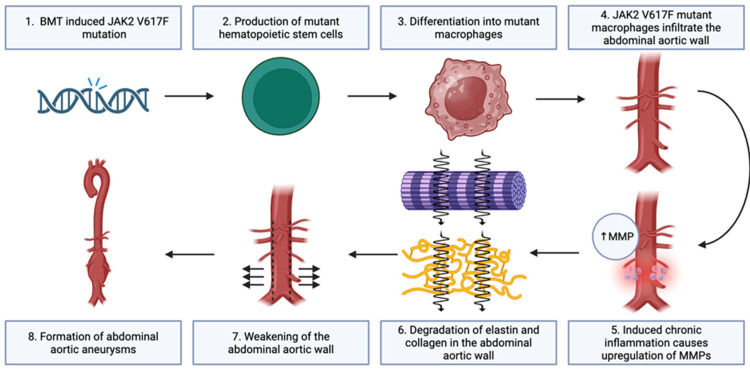
Schematic of *JAK2* V617F-mediated abdominal aortic aneurysm formation in ApoE–/– mice, adapted from [14].

Yokokawa used double transgenic *JAK2* V617F/CAG-EGFP bone marrow-transplanted (BMT) mice to model the *JAK2* V617F mutation. The researchers began by transplanting donor bone marrow cells from *JAK2* V617F transgenic mice or control wild-type (WT) into male irradiated apolipoprotein E-deficient (ApoE−/−) mice. Both the WT and *JAK2* V617F-modified mice were then crossed with CAG-EGFP mice to generate *JAK2* V617F/CAG-EGFP (experimental) and WT-GFP (control) strains. Five weeks after bone marrow transplantation, both *JAK2* V617F-mutant mice and WT mice were infused with 1900 ng/kg per min of angiotensin II (Ang II) or saline continuously for 4 weeks, with the goal of inducing aortic aneurysm formation. The researchers then monitored the mice for the presence of TAA and AAA, which were defined as an increase of ≥50% in aortic diameter compared with the baseline. No significant difference in thoracic aortic diameter was observed in both WT-BMT mice and *JAK2* V617F-BMT mice, even after Ang II infusion. However, *JAK2* V617F-BMT mice exhibited significant enlargement of the abdominal aorta, with 12 of the 26 mice (46.1%) developing AAA. MMP-2 and MMP-9 expression levels were significantly elevated in *JAK2* V617F-BMT mice after Ang II infusion. Additionally, there was a significant increase in CD45+ leukocytes, CD68+ macrophages, Ly6B.2+ neutrophils, and TER119+ erythrocytes in the abdominal arterial walls of *JAK2* V617F-BMT mice after Ang II infusion. Interleukin-1β (IL-1β) signaling has also been shown to play a central role in aneurysm development by promoting macrophage recruitment and increasing the expression of MMPs that degrade elastin and collagen within the aortic wall [25]. This inflammatory pathway aligns with the elevated cytokine signaling and MMP activity observed in *JAK2*-mutant models. These inflammatory findings are biologically consistent with the intrinsic activation of the JAK-STAT signaling pathway produced by the *JAK2* V617F mutation. Persistent activation of STAT transcription factors promotes the transcription of pro-inflammatory mediators and cytokines, which may further amplify vascular inflammation and matrix degradation [26]. Interaction between JAK-STAT signaling and other inflammatory pathways, including NF-κB, may further sustain immune cell activation within the vascular wall [27]. The inflammatory signaling framework is consistent with broader observations linking clonal hematopoiesis to accelerated atherosclerosis. Prior studies have demonstrated that somatic mutations associated with CHIP promote macrophage-driven vascular inflammation and atherosclerotic plaque development through increased cytokine production and immune cell activation [28,29,30,31]. These findings suggest that *JAK2*-mutant clonal hematopoiesis may contribute to aneurysm pathogenesis not only through direct inflammatory remodeling but also through the amplification of atherosclerotic inflammation. Based on the findings of the study, a schematic presentation of the proposed mechanism was developed (see Figure 1) [14].

Although the murine model provides valuable mechanistic insights, several limitations arise when attempting to align its findings with clinical data. First, the study used apolipoprotein E-deficient (ApoE−/−) mice, which are genetically predisposed to develop atherosclerosis [32]. Research has shown that atherosclerosis and pro-atherosclerotic genes are associated with the formation of AAA [33,34]. Given the results of the study, the ApoE−/− background likely impacted the results, as the mice only developed AAA. This contrasts with the clinical data presented above, which indicate that the *JAK2* V617F mutation is more strongly associated with TAA formation.

Another limitation is the angiotensin II (Ang II) infusion protocol. The researchers administered 1900 ng/kg per min continuously for 4 weeks, a supraphysiologic dose designed to overwhelm the vascular system of the mice [14]. While effective in inducing aneurysm formation, this approach does not accurately reflect the vascular stress of the average human individual with the *JAK2* V617F mutation, thereby jeopardizing clinical relevance. Furthermore, the ApoE–/– model combined with Ang II infusion has been shown to preferentially produce AAA, reflecting an inflammatory, atherosclerosis-driven AAA model rather than the medial degeneration typically seen in thoracic aneurysms [35]. Future studies using alternative experimental models, such as β-aminopropionitrile (BAPN)-based aneurysm models with or without Ang II infusion, may help to clarify whether *JAK2* V617F contributes to segment-specific aneurysm formation within the thoracic aorta.

The study by Al-Rifai also produced murine models with the goal of examining the mechanistic nature of the *JAK2* V617F mutation. The researchers modeled the *JAK2* V617F mutation with via four different types/groups of mutated cells: hematopoietic and endothelial cells, endothelial cells, hematopoietic cells, and myeloid cells [15]. The results of each murine model will be reviewed individually.

First, *JAK2* V617F-mutated hematopoietic and endothelial cell (HC-EC) mice developed MPNs, manifested in higher red blood cell, platelet, and leukocyte counts compared with controls. After 7 weeks, the mice exhibited aortic dilation in the descending thoracic and abdominal regions, accompanied by increased elastin degradation and MMP activity [15].

Second, in *JAK2* V617F-mutated endothelial cell (EC) mice, no MPNs or aortic dilation were observed [15].

Third, the hematopoietic expression of *JAK2* V617F caused the development of MPNs, elastin degradation, and aortic dilation [15].

Finally, the *JAK2* V617F mutation was induced in myeloid cells. *JAK2* V617F Myel mice developed MPNs with marked elastin and collagen degradation, as well as dilation of the ascending, descending, and abdominal aortas [15].

Taken together, these models demonstrate that *JAK2* V617F promotes aneurysm formation through hematopoietic and myeloid lineages. The mutation drives vascular tissue-resident macrophages toward a pro-inflammatory phenotype promoting aortic aneurysms.

An important distinction of the latter study is that aortic aneurysm formation occurred in the absence of a vascular stressor. In contrast, the pathophysiology of aortic aneurysms formation in humans is multifactorial, with vascular stress recognized as a major risk factor [36]. Clinical data indicate that the mean age of individuals with the *JAK2* V617F mutation and aortic aneurysms is approximately 65 years [14,15,16,17,18]. At this age, vascular stress is elevated due to age-related arterial stiffening, endothelial dysfunction, and other vascular alterations, all of which likely contribute to aortic aneurysm formation and progression [37,38].

## 4. Conclusions

The collective thrust from translational models, clinical datasets, and population-based cohorts highlights the positive association between the *JAK2* V617F somatic mutation and TAA formation, with a clear predominance in the ascending aorta. This regional specificity distinguishes *JAK2* V617F-driven aortopathy, suggesting that unique hematopoietic or hemodynamic mechanisms drive the vascular effects. Murine models suggest that *JAK2* V617F-mutant hematopoietic and myeloid lineages create a pro-inflammatory macrophage phenotype, which leads to MMP upregulation and elastin/collagen degradation. However, the downstream effects of these inflammatory signals on vascular smooth muscle cells (VSMCs) remain incompletely defined. VSMC dysfunction, phenotypic switching, and medial degeneration are well-recognized contributors to TAA pathogenesis [39]. Because most experimental studies examining *JAK2* driven vascular disease have focused primarily on hematopoietic and myeloid lineages, the role of vascular resident cells in this process remains insufficiently explored. Based on the currently available evidence, it remains unclear whether inflammatory mediators derived from *JAK2* V617F-mutant myeloid cells directly alter VSMC behavior or whether VSMCs primarily act as secondary responders to immune-driven matrix degradation. Future studies should investigate whether inflammatory signaling from *JAK2*-mutant immune cells promotes the maladaptive remodeling or the phenotypic switching of thoracic VSMCs. However, limitations arise in these germline-engineered models, including the use of ApoE−/− mice, and variable Angiotensin II use/dosage, complicating the translation of these mechanisms into human pathophysiology.

Across human studies, two consistent findings emerge. First, *JAK2* V617F has been associated with TAA, not AAA [14,15,16,17,18]. Second, VAF appears to modify risk, with higher burdens resulting in greater susceptibility to TAA. While population-based studies (UK-Biobank and DANCAVAS) demonstrate that *JAK2* V617F CHIP can predispose to TAA, even without overt hematologic malignancies, the majority of clinical cases were individuals with diagnosed MPNs [14,15,16,17,18]. Taken together, these findings suggest a broad range of TAA risk, from those individuals with *JAK2* V617F CHIP who have low VAF to those who have diagnosed MPNs and high VAF.

These observations raise several important clinical questions. Should all individuals with known MPNs, with high VAF, undergo thoracic imaging to screen for TAA? Echocardiography is radiation-free, safe, and relatively inexpensive. MR imaging is definitive and radiation-free, but costly. CT scan imposes a small radiation burden. The reviewed literature supports such screening, but further investigation of this relationship within institutional or population-specific cohorts is needed to determine its necessity. Second, should *JAK2* V617F CHIP carriers be considered for serial surveillance strategies, given their increased risk for TAA? One might argue affirmatively, although aneurysm growth is generally indolent, and a patient of advanced age may be unlikely to “sprout” a substantial aneurysm over the remaining lifespan. Due to the older age of these individuals, they may require thoracic imaging for other comorbidities, providing an opportunistic time to screen for TAA.

Several clinical and mechanistic gaps remain. The biological mechanism explaining the ascending TAA specificity of *JAK2* V617F remains unclear, warranting further investigation in animal models that better recapitulate the human disease. Our group has long emphasized that ascending and descending TAAs are different diseases [19]. For further animal model investigation, a study using genetically engineered *JAK2* V617F hematopoietic mice exposed to more moderate Angiotensin II infusions (500–1000 ng/kg/min) may provide a more physiologically relevant and accurate representation of the clinical phenotypes associated with *JAK2* V617F. Additionally, the relationship between increasing *JAK2* V617F VAF and TAA formation warrants further analysis to more accurately assess whether increased VAF consistently corresponds to greater aneurysm formation risk. Finally, a study examining the relationship between VAF and aneurysm growth rate could yield valuable insights into disease progression and risk stratification.

Currently, genetic sequencing identifies pathogenic germline variants in only 20% of TAA cases [40]. This figure reflects only known inherited mutations. The true proportion of genetically mediated TAA cases may be substantially higher once somatic variants such as *JAK2* V617F are considered. Continued investigation into the role of somatic mutations in TAA formation is therefore warranted.

## Figures and Tables

**Table 1:** Range of *JAK2* V617F VAF by MPN subtype. Data adapted from reference [8].

Type of MPN	*JAK2* V617F VAF
Essential Thrombocythemia (ET)	Low
Polycythemia Vera (PV)	Medium
Primary Myelofibrosis (PMF)	High

**Table 2:** Summary of the results from the identified studies: [14,15,16,17,18].

Study	Crucial Role of Hematopoietic *JAK2* V617F in the Development of Aortic Aneurysms	*JAK2* V617F Mutation Drives Vascular Resident Macrophages Toward a Pathogenic Phenotype and Promotes Dissecting Aortic Aneurysms	Increased Risk of Thoracic Aortic Aneurysms with *JAK2* V617F	Somatic Variants Acquired Later in Life Associated with Thoracic Aortic Aneurysms: *JAK2* V617F	Aortic Aneurysm Risk and Somatic *JAK2* V617F Variation: Insights from a Multicenter, Population-Based Cardiovascular Screening Study
Age (years)	68 ± 12	65 ± 14	60.6 ± 6.5	70	68 ± 4
Gender	46% Male	61% Male	56% Male	40% Male	90.9% Male
*N*	39	382	417,759	12,349	8056
*N* (w/reported MPN presence)	39	157	0	11	--
*N* (w/*JAK2* V617F)	39	382	118	12	570
Variant allele frequency (VAF)	51 ± 30%	--	33 ± 15%	11.2–20%	4.6% (Median)
*N* (w/TAA and *JAK2* V617F)	7	--	--	5	83
*N* (w/ascending TAA)	7	--	--	4	57
*N* (w/descending TAA)	0	--	--	1	26
*N* (w/AAA and *JAK2* V617F)	2	--	--	0	39

## Data Availability

No new data were created or analyzed in this study. Data sharing is not applicable to this article.

## Abbreviations

The following abbreviations are used in this manuscript:

TAA	Thoracic aortic aneurysm
AAA	Abdominal aortic aneurysm
VAF	Variant allele frequency
MPN	Myeloproliferative neoplasm
CHIP	Clonal hematopoiesis of intermediate potential
MMP	Matrix metalloproteinase
ApoE−/−	Apolipoprotein E-deficient
BMT	Bone marrow transplant
Ang II	Angiotensin II
